# Data and Service Security of GNSS Sensors Integrated with Cryptographic Module

**DOI:** 10.3390/mi14020454

**Published:** 2023-02-15

**Authors:** Changhui Xu, Jingkui Zhang, Zhiyou Zhang, Jianning Hou, Xujie Wen

**Affiliations:** 1 State Key Laboratory of Satellite Navigation System and Equipment Technology, Shijiazhuang 050081, China; 2 Key Laboratory of Surveying and Mapping Science and Geospatial Information Technology of MNR, Chinese Academy of Surveying & Mapping, Beijing 100036, China; 3 Geology Surveying and Mapping Institute of Guangdong, Guangzhou 510800, China; 4 National Engineering Research Center of Cryptography Science and Technology, Beijing 100043, China

**Keywords:** GNSS, cryptography, data security, service security, integrity protection, authorization, confidentiality

## Abstract

Navigation and positioning are of increasing importance because they are becoming a new form of infrastructure. To ensure both development and security, this study designed a technical innovation structure to upgrade the GNSS (Global Navigation Satellite System) data transmission and real-time differential correction service system and proposed a new multiple cryptographic fusion algorithm to achieve the encryption and decryption of GNSS data and services. First, a GNSS station encrypts GNSS data with an encryption key and obtains a public key from a GNSS data center to encrypt the GNSS data encryption key. After that, identity authentication of a GNSS station is carried out, and an SSL VPN is established between the GNSS station and a GNSS data center before GNSS data are transmitted to the GNSS data center. Then, the GNSS data center decrypts the received GNSS data. The process of an intelligent terminal for real-time differential corrections is similar to that of the GNSS station and the GNSS data center. A GNSS sensor integrated with a cryptographic module was developed to validate the structure in an open environment. The results showed that the developed GNSS sensor was successful in encrypting the data, and the GNSS data center was able to decrypt the data correctly. For the performance test, a cryptography server was able support the requirements of GNSS applications. However, a cryptography server was optimal in supporting 40~50 GNSS stations simultaneously, whereas a cluster was suggested to be configured if the number of GNSS stations was more than 60. In conclusion, the method was able to ensure the validity, confidentiality, integrity, and non-repudiation of GNSS data and services. The proposed upgrading technology was suitable for coordinating GNSS development and security.

## 1. Introduction

Surveying and mapping geographical information are important parts of national basic data, the security of which is a matter of national security. Generally, this information includes three types of data. Secret data are used for secret-related people, public data are used for every person, and controlled data between these two types of data are used for private people. Global navigation satellite system (GNSS) reference stations (also named Continuously Operating Reference Station, CORS) are the key infrastructure of surveying and mapping geographical information for high-precision navigation and positioning concerning these three types of data and are thus paid more attention. GNSS reference stations are the fixed stations on the ground that receive long-term continuous measured satellite signals; they transmit to the data center, in real time or at regular times, via communication facilities. Besides GNSS reference stations, GNSS receivers are applied for deformation monitoring, including landslide monitoring, oil rig monitoring, bridge monitoring, and high precision location services, including intelligent terminals, wearable devices, and special vehicles. Traditionally, we only pay attention to GNSS reference stations, the data of which are transmitted by private networks for security. However, the security of GNSS monitoring stations is not focused on because they are generally distributed over a small area. With the increasing importance of data security and individual privacy, applications of GNSS receivers should be controlled; otherwise, they will cause security hazards.

GNSS monitoring data for landslides, bridges, and oil rigs are transmitted on the internet, by tradition. Landslide monitoring usually sets several GNSS monitoring stations on a side slope or geological hazard susceptibility slope to obtain GNSS observations for deformation and sedimentation monitoring [1,2]. Bridge monitoring uses three to more than ten stations on different parts of a bridge to monitor its dynamic deformation and extract its structural health characteristics [3,4]. Oil rig monitoring also uses several GNSS stations to analyze a platform’s shaking trend and structural characteristics for offshore platform security [5,6,7]. The risk of these data is small, because the monitoring stations are usually distributed over a limited area. However, with the development of the internet, big data, and artificial intelligence, if GNSS monitoring data in a large area, even nationwide, are illegally collected, these data can be used to analyze national unstable regions, which can cause military or other risks.

GNSS high-precision location services are widely used for various fields. To obtain the high-precision coordinates, there are two main modes. One is Precise Point Positioning (PPP), which uses station observations to obtain the centimeter-level coordinates of the station with global precise orbit and clock corrections [8,9,10]. The other mode is Real Time Kinematic (RTK), or network RTK, which uses a reference station or reference network stations to eliminate common errors between a solution station and the reference stations so that real-time centimeter-level coordinates can be determined [11]. However, PPP has a long convergence, and RTK is limited by the distance between a rover station and a reference station. To optimize the advantages and complement the disadvantages of PPP and RTK, PPP-RTK has been developed, which can obtain centimeter-level coordinates without a reference station [12,13,14,15]. Therefore, GNSSs are increasing for location-based services, natural source surveying and monitoring, and intelligent terminals because of their high precision. Especially, with the ability of GNSS observations’ output in mobile phones, high-precision positioning of mobile phones is rapidly being investigated to provide precision positioning for the public [16,17]. All researchers focus on how to obtain high-precision positioning, and the techniques and applications have made great improvements. However, the security of GNSS data and service is not paid close attention. High-precision positions, especially individual and special vehicle trajectories, are private, which may cause personal risks, such as using individual information for illegal actions. It is necessary to develop a new method to protect information when these applications are popularized.

To improve the GNSS data security, there are mainly two types of solutions. One solution is based on algorithms. The multi-constellation receiver has better counter spoofing and replaying attacks, an approach of the clustering-based solution separation algorithm which was developed with the advantage of more redundant measurements [18]. By validating absolute and relative times of cross-checking the GNSS time, and time from other available sources, a time offset validation method is proposed to detect if the GNSS receiver was attacked or not [19]. The other solution for GNSS protection is cryptography. Panagiotis (2008) put forward the fusion of cryptography and GNSS in the future [20], and his team conducted some research about the analysis of attacks and countermeasures on cryptographically protected or unprotected GNSS signals [20,21]. At the same time, some studies are on the way, such as cryptographic authentication of the GPS navigation message, spoofing attacks against cryptographically secured GNSS signals of a hypothesis test, and cryptographic integrity of the navigation message [22,23,24]. A type of GNSS reference station (P5E) of CHC Navigation supports the HTTPs encryption. This study focused on security of GNSS data transmission from a receiver to a data center and a real-time differential correction service. We design a whole protection framework of GNSS data transmission and a real-time differential service with the cryptography technique to ensure the validity, confidentiality, integrity, and non-repudiation of GNSS data and services. At the same time, we improved a fusion algorithm based on multiple cryptographic algorithms for protection of the data source, encryption key, and transmission. Moreover, we also develop a new GNSS receiver integrated with a cryptographic module and validate the feasibility with an open environment.

## 2. Security Risk Nodes of GNSS Data and Service

**Figure 1:**
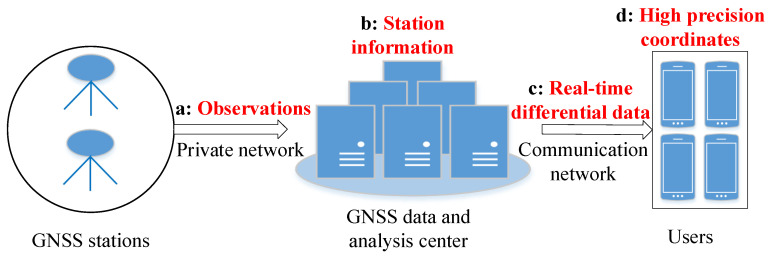
Security hazard nodes of GNSS data transmission and service.

Observation and navigation files of GNSS stations are free to download with a time delay by ftp or http in some countries of the world. At the same time, the International GNSS service (IGS) also provides the real-time service (RTS) correction streams for free to the broadcast ephemeris after registration. However, some information about GNSS reference stations is controlled for use in China to improve GNSS data security. This information includes four nodes from GNSS observation transmission to service, as shown in Figure 1.

- A GNSS receiver receives signals from GNSS satellites and transmits them to the GNSS data center. These signals can be transformed into GNSS observations such as pseudorange, carrier phase, and Doppler shift, which are controlled for use.
- Some GNSS CORS station information is controlled, except the secret data of original reference station coordinates and reference station network observations. This CORS station information is mainly the station name, type, level, location, receiver type, and so on.
- Real-time differential data are for two types of service. RTS combination products consisting of GNSS satellite orbit and clock corrections are for precise point positioning and real-time correction data for RTK or network RTK.
- All controlled data are used after verification and registration. Especially, service better than 1 m accuracy is in a higher controlled level.

## 3. Upgrading Structure of GNSS Integrated with Commercial Cryptography

It is of great significance to use commercial cryptography for presenting security hazards, so that the data are protected from stealing and tampering. To improve the security protection, we have to construct a basic commercial cryptography infrastructure, which consists of a cooperative signature system, digital certification identification system, key management system, cryptography management system, cryptography server, and a virtual private networks (VPN) server. The commercial cryptography infrastructure provides data encryption, data decryption, identical authentication, a virtual channel for data transmission, integrity protection, a collaborative signature, and key management for GNSS data transmission, storage, and service.

**Figure 2:**
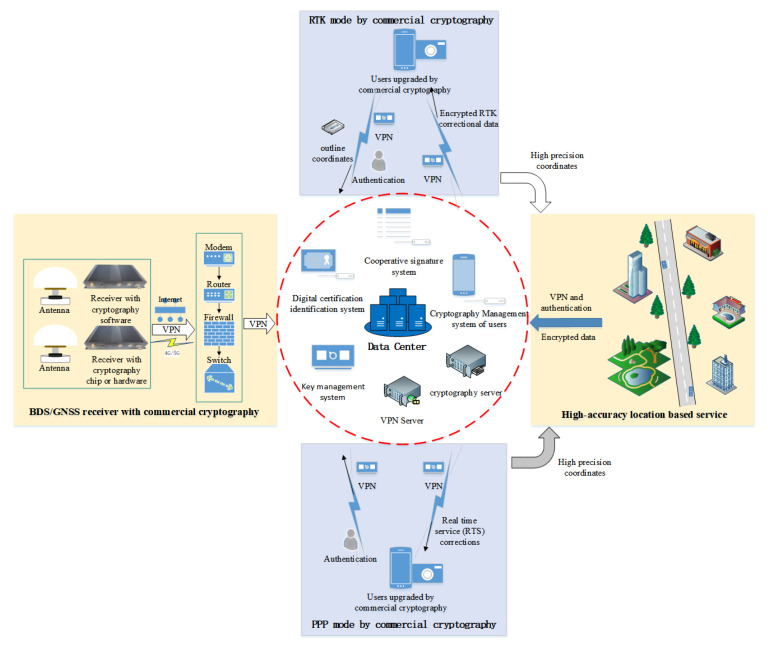
Commercial cryptography updating plan.

For the support of the commercial cryptography infrastructure, we proposed a plan of GNSS data security protection, as shown in Figure 2. The plan included the whole chain of GNSS data transmission, processing, storage, and a real-time differential correction service. In the process of GNSS data transmission from a GNSS receiver to a GNSS data center, the identification of the GNSS data center is authorized, the GNSS data are encrypted, and a virtual channel of GNSS data transmission is established. When the GNSS data are valued and stored, the signature is verified first, and then the GNSS data center is decrypted for usage and encrypted again for storage. At the same time, data integrity and the system log are protected. 

There are two types of service modes for high precision positioning to increase the GNSS data value. PPP can reach centimeter positioning precision using the RTS combination products with convergence over a period of time. RTK or network RTK uses correctional data of a single station or regional stations to obtain centimeter positioning precision within a minute. Therefore, we proposed two different commercial cryptography updating plans for these two application modes. A virtual channel and the authentication and integrity of PPP are required, while RTK needs a data encryption besides a virtual channel, authentication, and integrity. When we receive the high-precision coordinates of a position with these modes, they will become the controlled information if the coordinates are higher than some level or combined with geographic attribute information. This information is not suitable to transmit directly by a network. However, it is transmitted by the network after the commercial cryptography updating of security, integrity, and the virtual channel.

## 4. GNSS Data Security Protection Integrating Multiple Cryptographic Algorithms

Domestic commercial cryptography has many encryption algorithms, such as SM2, SM3, SM4, and SM9. The combination of SM2 and SM4 is used for the GNSS data security protection, where SM4 is to encrypt the GNSS RTCM data and SM2 is to encrypt the encryption key.

**Figure 3:**
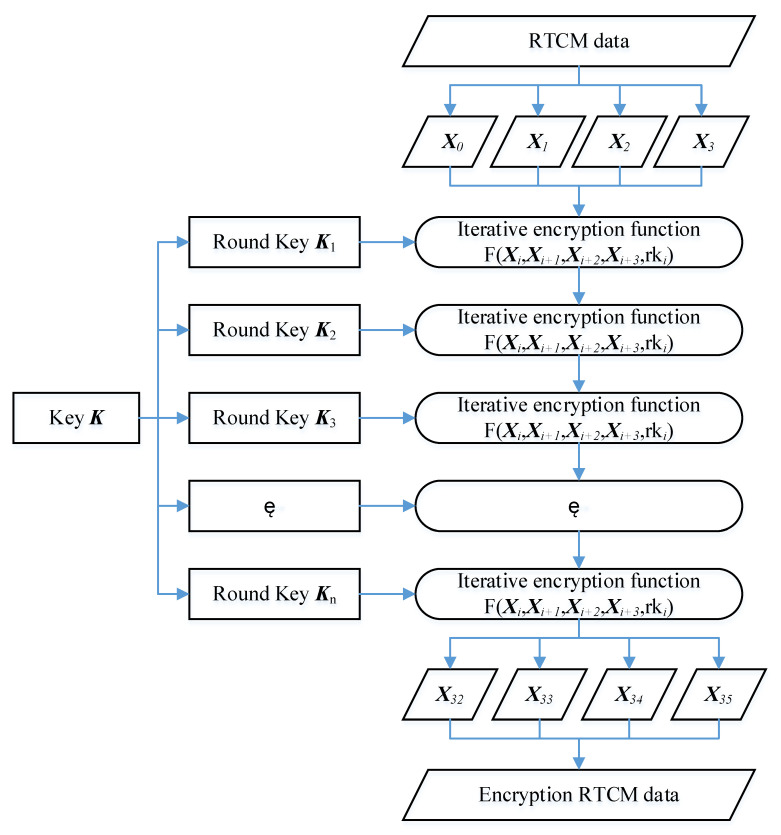
Iterative encryption of RTCM data.

GNSS RTCM data encryption uses an SM4 algorithm that encrypts and decrypts GNSS RTCM data with the same key so that it has high efficiency and fast encryption and decryption, with low costs and resources. However, the disadvantages of SM4 are the transmission, storage, and management of the cipher. To improve the security of the SM4 cipher, truly random numbers are generated regularly as the GNSS RTCM data encryption key by the cryptographic module. The SM4 algorithm is a block encryption algorithm with a block length and key length of 128 bits. It uses a nonlinear iterative structure of 32 rounds operated by nonequilibrium Feistel, with the same algorithm structure for encryption and decryption, and is easy to implement in hardware because of the same circuit topology. The iterative encryption algorithm is given in Figure 3.

GNSS RTCM data are to be encrypted. They are divided into four blocks consisting of 128 bits for each block, denoted as (*X*_0_, *X*_1_, *X*_2_, *X*_3_). Suppose (*Y*_0_, *Y*_1_, *Y*_2_, *Y*_3_) are the output encryption RTCM data; the encryption method is as follows:

(*Y*_0_, *Y*_1_, *Y*_2_, *Y*_3_) = *R*(*X*_32_, *X*_33_, *X*_34_, *X*_35_) = (*X*_35_, *X*_34_, *X*_33_, *X*_32_)

where *R* is an inverse transformation function. *X****_i_*** can be expressed as follows:

*X**_i_*****_+ 4_ =***X**_i_***⊕*T****’***(*X_i_*_+ 1_⊕*X_i_*_+ 2_⊕*X_i_*_+ 3_⊕*rk_i_*)  *i* = 0, 1, 2, …, 31,

where *rki* is the round key generated by encryption cipher, which can be expressed as the following:

*rk_i_* = *K_i_*_+ 4_ = *K_i_*⊕*T*(*K_i_*_+ 1_⊕*K_i_*_+ 2_⊕*K_i_*_+ 3_⊕*CK_i_*)

where *CK*_i_ is 32 constants of 32 bits; *K_i_* is determined by the xor operation between the cipher and constant, both of 128 bits; and *T* and *T****’*** can be found in [25].

Truly random numbers generated by the cryptographic module improve the security of the encryption key, but it is not safe if the truly random number is open to the internet. To protect the truly random number, an SM2 algorithm is used. SM2 is an elliptic curve public key cryptography algorithm that uses different keys for data sending and receiving to encrypt and decrypt the encryption RTCM data. The GNSS data center generates a pair of public and private keys, in which the public key is sent to the GNSS station receiver or other data-collecting device for encrypting the data and the private key stored in the GNSS data center for decrypting the data. Compared to SM4, SM2 is a more complicated algorithm with lower encryption and decryption velocities and higher security.

According to the SM2 encryption algorithm, some public parameters are agreed to by both the data center and receiver. These parameters include a prime number *p*, an elliptic curve defined on finite field *E*, and an *n*th basic point on the elliptic curve *G*. If (*x*_1_, *y*_1_) and (*x*_2_, *y*_2_) are two points on the elliptic curve, and *k* is a truly random number (*k* ∈ [1, *n* − 1]), a new point *C*_1_ and a public key *P* can be obtained by the following:

*C*_1_ = *kG* = (*x*_1_, *y*_1_)

*kP* = (*x*_2_, *y*_2_)

Then, the encryption key *C* is obtained by joining three parameters, given a random key *M* and the length *mlen*:

*C* = *C*_1_‖*C*_2_‖*C*_3_

where *C*_2_ = *M*⊕*KDF*(*x*_2_‖*y*_2_, *mlen*) and *C*_3_ = *H*(*x*_2_‖*M*‖*y*_2_); *KDF*(*x*_2_‖*y*_2_, *mlen*) is a key derivation function; and *H* is a compliant hash function.

## 5. Multiple Cryptographic Algorithms for GNSS Encryption and Decryption

**Figure 4:**
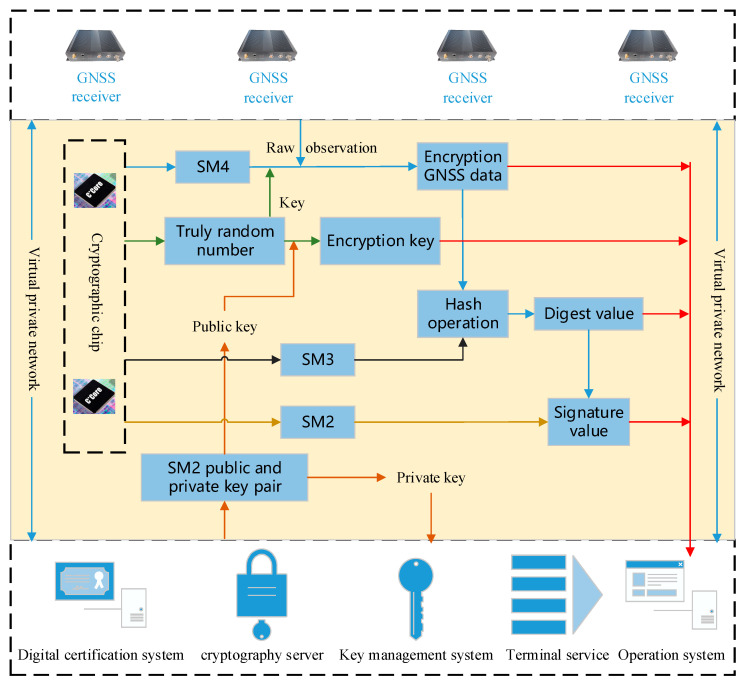
The flow chart of GNSS RTCM data encryption.

A GNSS receiver can be used in a GNSS reference station, a low-cost GNSS monitoring station, and an intelligent terminal. It is different among these applications because of the diversity of hardware and operation systems. Therefore, a customized encryption plan is necessary for these hardware and operation systems. For example, the SSL protocol based on a Virtual Private Network (SSL VPN) can be used for the Linux operation system in a GNSS reference station, while it is not adaptive for an ARM operation system in a low-cost GNSS monitoring station; however, it can be developed with that requirement. We tried to give a general encryption plan for GNSS RTCM data transmission (Figure 4), which could be changed with the needs of practical conditions. The encryption method of GNSS data transmission was expressed as Algorithm 1.
**Algorithm** **1**: Encryption steps of GNSS data transmission**     Input:** GNSS data, a digital certificate from a GNSS data center
**      Output:** the encrypted GNSS RTCM data, the encryption key, the digest value, and the signed inspection certificate**      if** identical authentication pass **then**      An SSL VPN to be established between GNSS stations and the GNSS data center
       **if** SSL VPN established **then**
generating a truly random number by cryptographic module embedded in the GNSS development board as an encryption keyencrypting the GNSS data with the encryption key of an SM4 algorithm by cryptographic moduleextracting the public key from a digital certificateencrypting the encryption key with the public keycalculating the digest value of GNSS encryption data with an SM3 algorithm by the cryptographic modulesigning the digest value with an SM2 algorithm by the cryptographic module to obtain a signed inspection certification and identify the certification of GNSS stationsoutputting all data including the encrypted GNSS RTCM data, the encryption key, the digest value, and the signed inspection certificationtransmitting all data to the GNSS data center by the communication module embedded in the development board.
**        end if**
**    end if**

When the encryption GNSS RTCM data are received in the GNSS data center, the signed inspection certificate is firstly identified to adjust the credibility of the data transmission status. If it is not credible, the data are rejected because they may be unsafe. Otherwise, the encryption data key is decrypted to obtain the private key through the index of the SM2 private key. After that, GNSS RTCM data can be obtained using the SM4 algorithm to encrypt GNSS RTCM data. Finally, the digest value of GNSS RTCM data is calculated by the SM3 algorithm to compare with the transmitted digest value for the integrity test.

In the field of the GNSS real-time differential service, the method is similar to the GNSS data transmission plan. There are SSL VPN, SM2, SM3, and SM4 algorithms for encryption, decryption, and integrity protection. Compared to GNSS stations transmitting data to the GNSS data center, the difference is that the GNSS data center encrypts the real-time differential correction data and broadcasts to users through the SSL VPN. The users receive the encrypted real-time differential correction data and decrypt them for navigation and positioning.

## 6. Experimental Analysis

Cryptographic algorithms were firstly implemented in GNSS navigation and positioning. The process was mainly related to two parts: a GNSS station and a GNSS data center. To validate the feasibility of GNSS data encryption transmitting, we developed a GNSS receiver integrated with a cryptographic module (Section 6.1) and performed a real test in an open environment (Section 6.2). When the receiver collected different numbers of satellites, the GNSS data were not the same. At the same time, a GNSS center could receive the data from several stations to thousands of stations. Therefore, we tested the performance of a GNSS data center under the conditions of different sizes of GNSS data and different numbers of GNSS stations (Section 6.3).

### 6.1. Development of a GNSS Receiver Integrated with a Cryptographic Module

**Figure 5:**
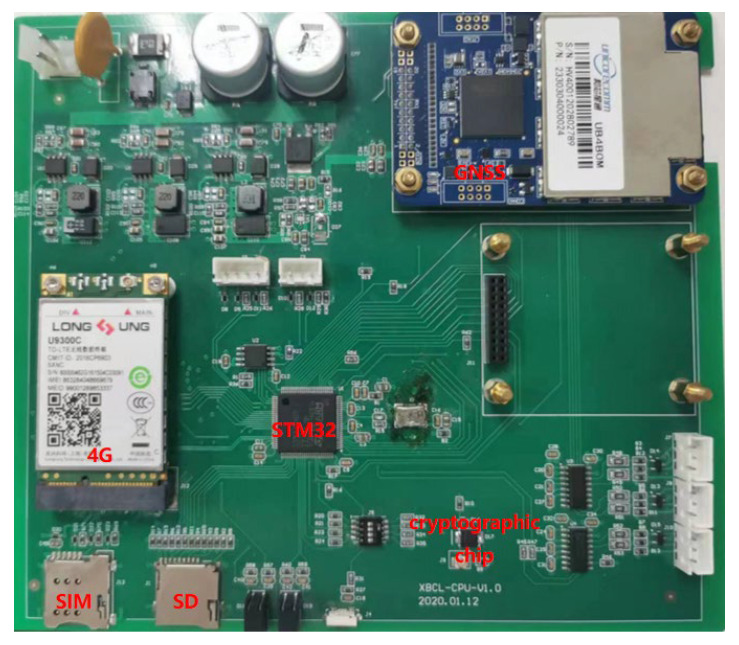
GNSS development board integrated with cryptographic module.

To test the multiple cryptographic algorithms for GNSS encryption and decryption, we developed a GNSS sensor with a GNSS development board, cryptographic module, and a 4G communication module (Figure 5). The GNSS development board was able to track multiple types of GNSS signals such as BDS B1I/B2I, GPS L1/L2, GLONASS L1/L2, Galieo E1/E5b, and QZSS L1/L2. It had 432 channels and supported global signals of BDS3. The cryptographic chip was CCM3310S-T, which supported multiple commercial cipher algorithms, such as SM2, SM3, and SM4. The highest system frequency of the cryptographic chip using 32 bits of RISC CPU reached up to 60 MHz. The 4G communication module was SLM730 C7B for all kinds of networks and multiple network systems such as TDD-LTE, WCDMA, CDMA, and GSM.

The experiment was designed to test our GNSS data encryption method using an RTK mode. We used a GNSS receiver as a reference station, and another GNSS receiver as a rover station. The encryption data of both the reference station and rover station were transmitted to a GNSS data server through a 4G network. The condition was suitable for RTK and network RTK applications.

### 6.2. Validation of Data Encryption in GNSS Receiver

The GNSS sensor integrated with cryptographic technology above was firstly developed to protect the data and service security. The GNSS sensor encrypted GNSS RTCM data, and the GNSS data center received and decrypted the data successfully, which ensured that the technical plan was feasible. At the same time, we needed to assess the performance of the upgraded GNSS sensor to confirm that they satisfied the time delay requirements in various applications. Therefore, the encryption time delay of GNSS RTCM data in the GNSS sensor and the decryption time delay of RTCM data and encryption time delay of real-time differential correction in the GNSS data center were operated.

**Figure 6:**
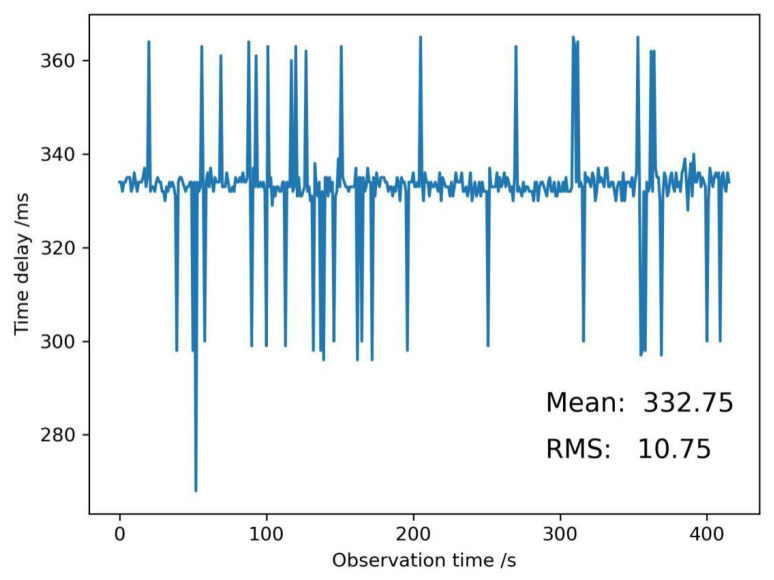
Encryption time delay of GNSS RTCM data in a GNSS sensor.

To identify if the data transmission and reception of the GNSS sensor after being integrated with the cryptographic module available, we set up a reference station and a rover station with the developed GNSS board to encrypt GNSS RTCM data in an open environment (Figure 6). Two stations observed GNSS satellites simultaneously. The time of the GNSS sensor receiving GNSS satellite signals was denoted as *t*_1_, and *t*_2_ was the time of GNSS satellite signals between the data being encrypted by the cryptographic module and transmitted to the GNSS data center by the 4G communication module. Then, the encryption time delay of the GNSS RTCM data in the GNSS sensor was expressed as follows:

*t* = *t*_2_ - *t*_1_

A series of encryption time delays of GNSS RTCM data were recorded (Figure 6). The mean encryption time delay value was 332.75 ms, and this was not a small time delay for GNSS applications. The reason for this was perhaps that the performance of the cryptographic module was to be improved, or the peripheral component interface express (PCIE) used in the paper was to be changed. The root mean square value (RMS) was 10.75 ms, which can be acceptable.

Encryption GNSS RTCM data were transmitted from a GNSS receiver to a GNSS data center through a 4G network. Every epoch’s data were different because of the different satellite systems and satellite numbers. A CAT4 communication module could transmit up to 750 bytes each frame, with a 50 mbps uplink data transmission rate. When encryption GNSS RTCM data were more than 750 bytes, the GNSS RTCM data were divided into two or more frames for transmission. After commercial cryptography updating, the average transmission time was increased by 2%, which could be ignored in GNSS applications.

### 6.3. Simulation Encryption and Decryption Concurrent Validation of a GNSS Data Center

When the GNSS data center received the GNSS RTCM data from a GNSS station, the GNSS data center decrypted the encryption GNSS RTCM data for various applications and encrypted them again for storage by calling a cryptography server. Therefore, if there were huge GNSS stations, the performance of the GNSS data center was of crucial importance to support real-time service.

Generally, processing GNSS stations are not the same for various services. If a GNSS is used for deformation monitoring, bridge monitoring, and dam monitoring, there would be 3~10 GNSS stations. When a GNSS is used for a provincial real-time differential correction service, there would be 50~100 GNSS stations, which is dependent on the province area and topographical environment. As we know, GNSS PPP is a positioning mode, with the needs of precise satellite orbit and satellite clock products, which are generated by 100~150 GNSS stations. The other mode is network RTK, whose real-time differential corrections come from thousands of GNSS stations. The number of GNSS stations was selected from 5 to 150 to test the encryption performance, with 1000 GNSS stations as the maximum number due to limitations of the testing software.

At the same time, the size of transmitted GNSS RTCM data varied according to environmental conditions of the GNSS sensor. The size was perhaps less than 750 bits because of several visible satellites in the urban- and forest-sheltered environment, and more than 2048 bits in an open environment due to more satellites being available. Therefore, we simulated different sizes of GNSS RTCM data, from 704 bits to 5120 bits, which could cover the entire range of data.

**Figure 7:**
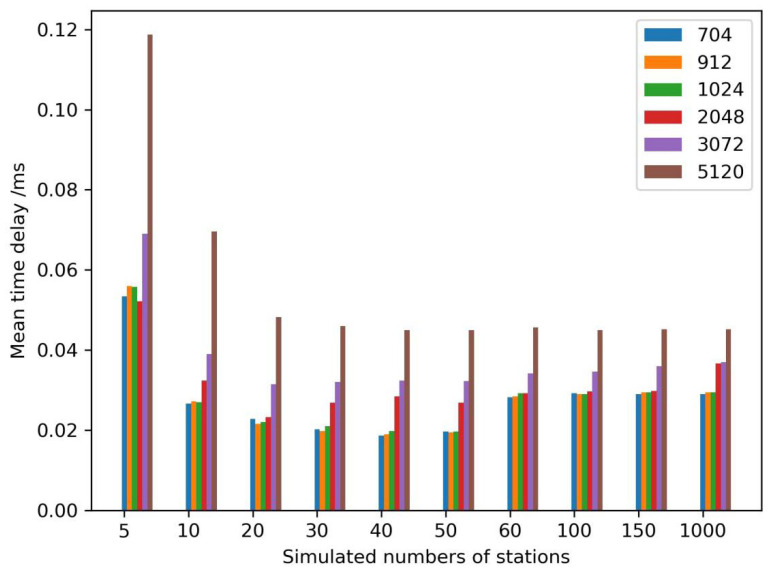
Encryption time delays of different simulated data volumes/bit.

A cryptography server from Westone Information Industry Inc. was selected to test the performance and stability. The recorded time was the difference between starting to decrypt the encryption GNSS RTCM data by calling the cryptography server and returning the plaintexts to the GNSS data center. The simulated results are shown in Figure 7. When the number of GNSS stations was five, the mean time delay was the largest among all simulated numbers of stations, and the optimal performance of the cryptography server was when the station number was equal to 40~50 stations. The performance of the cryptography server decreased with an increase in the station numbers when the station number was larger than 50. As a result, multiple cryptography servers were configured as a cluster if the station number was more than 50. Meanwhile, the performance was almost the same if the size of the data was 704 bits to 1024 bits. When the size was larger than 1024 bits, the mean time delay increased significantly at the optimal station numbers. When the number was between 60 and 150, the performance of 2048 bits was also the same as less than 2048 bits. The mean time delay increased with the growing size of the data, among all station numbers.

**Figure 8:**
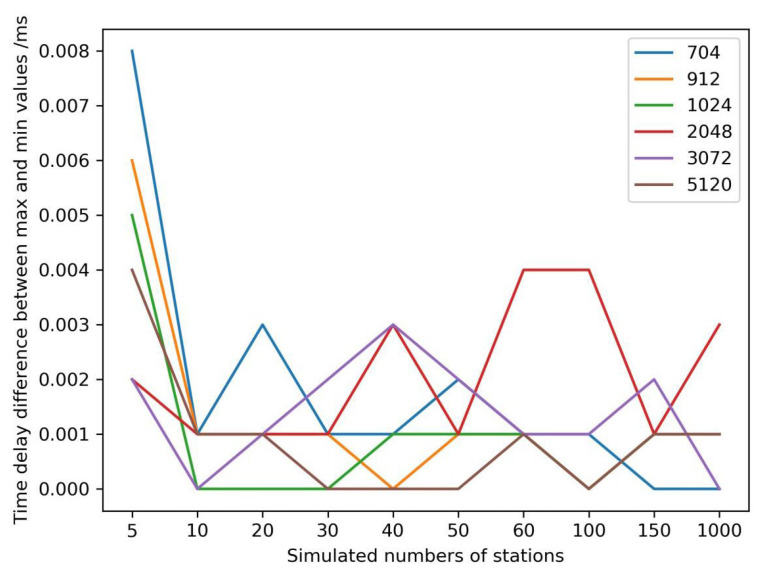
Encryption time delays of different simulated data volumes/bit.

The mean time delay could describe the whole encryption performance of the cryptography server. To evaluate the stability of the cryptography server, the time delay difference between the maximum and the minimum of the time delay series was determined (Figure 8). The maximum time delay difference was less than 0.008 ms, which would satisfy the requirements of GNSS applications.

**Figure 9:**
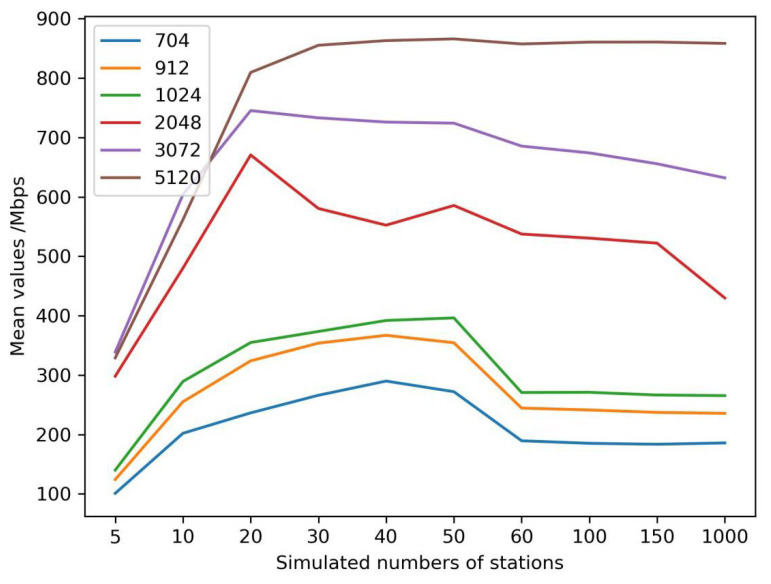
Encryption global rates of different simulated data volumes/bit.

The data rates of the cryptography server were fixed. We estimated an index of the million bits per second (mbps) to validate the optimal configuration of station numbers and data volumes (Figure 9). If the size of the data was more than 2048 bits, a cryptography server could support 20 simultaneous encryption and decryption stations; otherwise, the number of the stations was 50. A cluster of cryptography servers should be configured if the station number exceeds the numbers.

**Figure 10:**
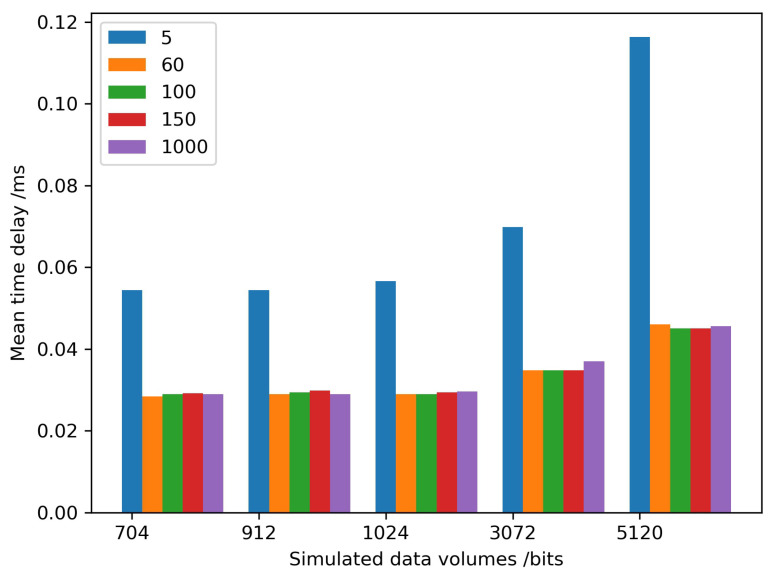
Decryption time delays of different simulated data volumes/bit.

The decryption performance of the same cryptography server was also tested according to different simulated stations and data volumes (Figure 10), which were the same as the encryption performance. When the station number was larger than 60, the decryption performance had little relationship with the data volumes; however, it was also not good if the stations were too few.

## 7. Conclusions

From the perspective of solving the contradiction between the development and security of GNSS navigation and positioning, this study designed an upgraded structure of GNSS data transmission and service using cryptographic technology to improve the security of GNSS RTCM data from a GNSS station to a GNSS data center, and of real-time differential corrections from a GNSS data center to an intelligent terminal. This research proposes a multiple cryptographic fusion algorithm to achieve the confidentiality protection of GNSS data and service.

The developed GNSS sensor with the cryptographic module in this study has a mean encryption time delay of 332.75 ms, which is a little long for GNSS applications to be improved further, hence cryptographic software is suggested for GNSS sensor integration now. At the same time, the time delay on internet transmission caused by added encryption data can be ignored. For a GNSS data center, the time cost is less than 0.04 ms when the data volumes are smaller than 5120 bits, and the stability of the cryptography server is about 0.008 ms. Therefore, the performance of the cryptography server is satisfactory for the encryption and decryption requirements of GNSS applications. However, a cryptography server is not suitable for many stations, such as for more than 60 stations. When a GNSS data center collects from more than 60 stations, this study suggests a cluster of cryptography servers to be configured according to each cryptography server with 40~50 stations.

The proposed technical structure and upgrading method are suitable not only for traditional reference data transmission and real-time differential correction service but also for low-cost GNSS applications with an ARM platform that are used for various monitoring receivers and high-precision location services, such as intelligent terminals, wearable devices, and special vehicles. Furthermore, other controlled data collections and services are also able to use the proposed structure to improve security, such as ocean data collection and transmission, and transportation data monitoring.

## Data Availability

Not applicable.
